# Myocarditis in Paediatric Patients: Unveiling the Progression to Dilated Cardiomyopathy and Heart Failure

**DOI:** 10.3390/jcdd3040031

**Published:** 2016-11-08

**Authors:** Inês Teixeira Farinha, Joana Oliveira Miranda

**Affiliations:** 1 Faculty of Medicine of Porto University, Porto 4200-319, Portugal; 2 Department of Paediatric Cardiology, Centro Hospitalar São João, Porto 4200-319, Portugal; joanam@gmail.com; 3 Department of Physiology and Cardiothoracic Surgery, Faculty of Medicine of Porto University, Porto 4200-319, Portugal

**Keywords:** myocarditis, dilated cardiomyopathy, heart failure, inflammation, viral myocarditis

## Abstract

Myocarditis is a challenging and potentially life-threatening disease associated with high morbidity in some paediatric patients, due to its ability to present as an acute and fulminant disease and to ultimately progress to dilated cardiomyopathy. It has been described as an inflammatory disease of the myocardium caused by diverse aetiologies. Viral infection is the most frequent cause of myocarditis in developed countries, but bacterial and protozoal infections or drug hypersensitivity may also be causative agents. The prompt diagnosis in paediatric patients is difficult, as the spectrum of clinical manifestation can range from no myocardial dysfunction to sudden cardiac death. Recent studies on myocarditis pathogenesis have revealed a triphasic nature of this disease, which influences the diagnostic and therapeutic strategies to adopt in each patient. Endomyocardial biopsy remains the gold standard for diagnosing myocarditis, and several non-invasive diagnostic tools can be used to support the diagnosis. Intravenous immunoglobulin has become part of routine practice in the treatment of myocarditis in paediatric patients at many centres, but its true effect on the cardiac function has been the target of many studies. The aim of this review is to approach the recently discovered facets of paediatric myocarditis regarding its progression to dilated cardiomyopathy.

## 1. Introduction

Myocarditis is a non-familial form of inflammatory heart muscle disease, in the absence of predominant acute or chronic ischaemia [1,2,3]. It is, by definition, an inflammation of the myocardium, which may also extend to the pericardium and endocardium [4]. Diagnosis is established by histological, immunologic and immunohistochemical criteria, as it was defined by the World Health Organization (WHO)/International Society and Federation of Cardiology (ISFC), in 1995 [5]. It is an important cause of morbidity and mortality in children, due to its association with cardiac dysfunction and dilated cardiomyopathy (DCM), which may represent the chronic phase of the disease [5,6].

## 2. Aetiology

Acute myocarditis has multiple causes, including viruses, protozoa, bacteria, fungi, toxins, drugs, metabolic abnormalities, hypersensitivity reactions and systemic autoimmune diseases [7]. Often the underlying agent causing the disease is not identified [4].

### 2.1. Infections

More than 50% of paediatric acute myocarditises in Western Europe and North America are caused by cardiotropic viruses [8,9]. The pattern of viral pathogens associated with myocarditis has evolved over the last 20 years [10]. Enteroviruses (particularly coxsackieviruses) have traditionally been considered the most frequent viral agents in myocarditis and DCM [11,12]. More recently, new molecular techniques have revealed that the most commonly detected viral agents were Parvovirus B19 and human herpesvirus type 6 [12,13,14]. Other previously unrecognized viruses associated with myocarditis with varying degrees of frequency include adenovirus, cytomegalovirus, Epstein–Barr, hepatitis C, herpes simplex type 2, influenza and parainfluenza viruses [9,11,13,15,16,17,18,19]. In 2010, Bratincsák et al. reported three cases of fulminant myocarditis in a paediatric population associated with the pandemic H1N1 influenza A virus infection, and it is hypothesized that this strain may be more associated with severe forms of myocarditis than other influenza strains [20]. Human immunodeficiency virus (HIV) has also been associated with myocarditis, which is often asymptomatic [21]. HIV myocarditis may cause both systolic and diastolic dysfunction in paediatric patients [22]. Some agents are thought to be responsible for the initiation of these processes and the presence of viral nucleic acid in the myocardium, a common finding in HIV+ children, may be one of them [23]. Infection of myocytes with other agents, like fungi, parasites or other viruses other than HIV may be associated with the development of DCM and congestive heart failure [23,24]. Some drugs used in highly active antiretroviral therapy (HAART) regimens, like Zidovudine, are cardiotoxic themselves [22].

Bacteria and other infectious causes, such as protozoa, fungi, and parasites, have been known to cause myocarditis far less commonly than viruses [25]. Toxin-producing bacteria, including *Clostridium* and *Diphtheria*, can cause severe myocardial damage, the latter causing myocarditis in countries without widespread immunization [21,26]. Lyme disease, caused by the spirochete *Borrelia burgdorferi*, can also result in acute myocarditis [7]. However, it is considered the least common manifestation of early disseminated Lyme borreliosis in children and progression to DCM is rare [7,27]. In 2009, Costello et al. reviewed 207 cases of children with early disseminated Lyme disease and found that 16% had myocarditis, 42% of whom had advanced atrioventricular heart block, including 27% with third-degree heart block [28]. In Latin-American countries, Chagas disease, which results from an infection due to the protozoan *Trypanosoma cruzi*, is a common cause of acute myocarditis and DCM [10,29]. In recent years, as a consequence of effective control of the transmission of the protozoan, acute Chagas myocarditis has reduced in Brazil [7].

### 2.2. Hypersensitivity to Drugs and Other Substances

Drugs may also cause myocardial inflammation via a direct cardiotoxic effect or by inducing hypersensitivity reactions [4]. Hypersensitivity myocarditis is a rare form of inflammatory disease of the myocardium [30]. The molecular basis is poorly understood, but it is thought to be a hypersensitivity reaction to haptens from the cardiotoxic drug [31]. This diagnosis should always be suspected when clinical presentation occurs in a setting consistent with drug allergy because of its excellent prognosis after withdrawal of the causative toxin [32]. Some agents have been reported to be implicated in hypersensitivity myocarditis in paediatric patients. These include chemotherapeutic drugs, such as anthracyclines, dermatology drugs, like dapsone, and also tetanus and smallpox vaccines [30,33,34,35].

These drug-induced hypersensitivity responses are often associated with an eosinophilic myocarditis, which is usually revealed after an endomyocardial biopsy (EMB) [21]. In this situation, withdrawal of the offending medication may not always be enough and corticosteroid therapy may be needed [36].

## 3. Myocarditis: A Triphasic Disease Process

**Figure 1:**
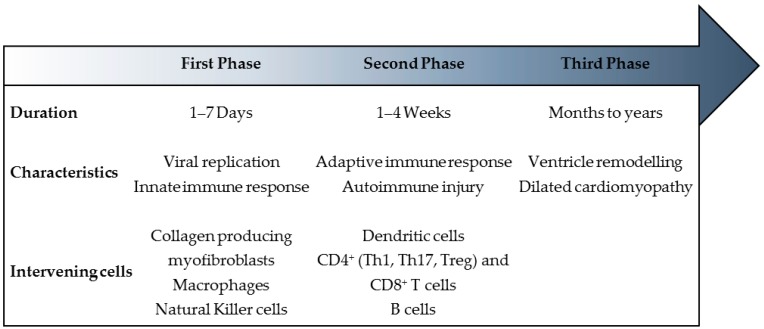
Myocarditis: A triphasic disease. In recent years, myocarditis has been set in a framework of three successive and distinct phases with undefined borders. For each phase, average duration (regarding the case of coxsackievirus-mediated myocarditis), characteristics and physiopathological intervenients are indicated. CD, cluster of differentiation; Th1, T helper 1; Th17, T helper 17; Treg, regulatory T.

Since the majority of cases of myocarditis result from a viral infection, it has long been studied in experimental murine models as a virus-induced autoimmune disease, which may progress to gradual myocardial dilatation [3,37]. Myocarditis progresses through stages with distinct processes and manifestations. Thus, it has been helpful to set it in the structure of a continuum of three chronologically successive phases of disease (Figure 1). For each phase, pathogenesis, diagnosis, and treatment differ significantly [37,38].

The three-phased course starts with the first phase, which is an induction stage where the virus plays a major role in the initial insult to the myocardium [6,38]. The entry and active replication of the virus is followed by cardiomyocyte lysis and activation of the host innate immune responses. In most patients, the immune system downregulates to a resting state after viral proliferation is brought under control and they recover without significant sequelae [3,37]. Thus, the initial insult often goes unnoticed [38].

If host immune activation persists despite elimination of the virus, the second phase develops as a result of both adaptive immune and autoimmune responses [38]. The activation of antigen-specific immunity involving T cells, B cells and antibody production contributes to additional myocardial injury with deleterious effects [3,10]. Development of autoantibodies can be induced by molecular mimicry between myocardial antigens and viral peptides [10,38]. Clinically overt congestive heart failure may develop in this phase [38]. In the majority of patients, the virus is eliminated and the immune reaction is downregulated [3].

However, a subset of patients progresses to a third phase in which the inflammatory processes persist and the disease develops into DCM and it may progress despite the ending of the first phases [3,38]. Viral myocarditis has become recognized as an important cause of DCM in children. Miranda et al. performed a retrospective review of 61 paediatric patients (37 female; 24 male) diagnosed with DCM and found that viral myocarditis was the aetiology in 18% of cases [39].

## 4. Pathogenic Mechanisms

The causal connection between myocarditis and its progression to DCM and heart failure is not completely understood but has been hypothesized for many years [40]. Different studies using murine models of myocarditis have contributed to what is currently known about the pathogenesis of the disease [4]. Viral mediated cardiomyocyte injury, autoimmunity and persistent viral infection are three theories assumed to explain this process [41].

The disease begins with viral entry into cardiomyocytes [21]. Specific receptors have been identified on human myocytes, such as the Coxsackie-adenoviral receptor (CAR) for Coxsackieviruses of group B and some adenoviruses, and the erythrocyte P-antigen cellular receptor for Parvovirus B19 [42,43,44,45,46]. Replicating viruses in the absence of an immune response causes myocytolysis, which is the primary lesion responsible for focal necrosis and inflammation of the myocardium [40,47]. Subsequently, innate and adaptive immune responses are activated [7,38]. Many host pro-inflammatory mediators are upregulated in an attempt to limit viral replication, including cytokines such as tumour necrosis factor-α (TNF-α), interleukin (IL)-1 and IL-6, nitric oxide, macrophages and natural killer (NK) cells [21,48]. Additionally, T lymphocyte (CD4+ and CD8+) activation was described to induce cytokine production as well as apoptosis of targeted cells mediated by perforins, serine esterases and the Fas ligand/Fas receptor pathway [49]. Even though these mechanisms are an essential path to recovery, their cumulative effect is harmful since they also enhance myocardial tissue injury, therefore contributing to the loss of virus-harbouring myocytes [21,38,50]. The decrease in the number of contractile units and their inability to regenerate may lead to long-term remodelling and a clinical picture consistent with DCM [6,7].

Autoimmunity has also been recognized as having an important role in the progression of viral myocarditis [41]. Secondarily to myocyte necrosis comes the exposure of heart muscle antigens [51]. Questions remain whether this mechanism is the only one triggering the autoimmune disease or if molecular mimicry may also play a part [41]. When activated B lymphocytes produce antibodies that cross-react with these antigens the autoimmune response begins and viral myocarditis progresses [7,41]. Anti-sarcolemmal, anti-myosin alpha and beta heavy chains, anti-mitochondrial proteins, and anti-β1 adrenergic receptor antibodies have all been identified in patients with DCM [7,52].

According to Kuhl et al., persistent viral genome can be found in 67.4% of adult patients with DCM, which is most likely caused by incomplete clearance of the pathogen [4,53]. The exact role of a persistent cardiac infection in the development of DCM in paediatric patients has not been defined yet [38]. It is hypothesized that continuous or intermittent virus replication may result in a chronic myocyte damage and autoimmune injury [21,41].

## 5. Clinical Presentation

Clinical manifestation of the disease in children has a variable spectrum, ranging from non-specific systemic symptoms with no haemodynamic consequences (fever, myalgia, palpitations or dyspnoea) to congestive heart failure, ventricular dysfunction, shock and life-threatening ventricular arrhythmias, which consequently can lead to sudden cardiac death [6,7,54]. Syncope and chest pain may also be presenting complaints [10]. The latter can mimic adult myocardial infarction with anterior chest pressure pain radiating to the neck and arms and is typically seen in younger patients with few cardiac risk factors [2,4,55]. This acute myocardial infarction-like syndrome can be associated with electrocardiographic changes, such as ST segment elevation, and elevated biomarkers of myocardial cell damage (creatine kinase and/or troponin I), in a context of normal coronary arteriography [2,7,56].

Many patients may present with mild unspecific prodromal symptoms typical of a viral illness, such as shortness of breath, vomiting, anorexia, abdominal pain, diarrhoea, fever, myalgia, lethargy, syncope or seizures [4,7,57,58]. A history of respiratory tract infection or gastroenteritis may precede the onset of myocarditis by several days to a few weeks [7]. In 2009, Durani et al. performed a retrospective review of the presenting symptoms in patients ultimately diagnosed with myocarditis, and the most frequently described were shortness of breath (69%), vomiting (48%), poor feeding (40%), upper respiratory symptoms (39%), fever (36%), and lethargy (36%) [59].

Clinical features also vary according to age. Infants may present with nonspecific symptoms like anxiousness, malaise, fever, poor appetite, tachypnoea, tachycardia, and cyanosis [21]. Children greater than two years of age may also complain of chest pain, shortness of breath, abdominal pain, exercise intolerance, myalgia, arthralgia, fatigue, palpitations, cough or oedema [21,41,60].

The severity of symptoms is dependent on the age of the child [21]. Newborns and infants are often more severely affected and, in contrast to older children and adults, they are more likely to present with circulatory shock and acute DCM and may require advanced circulatory and respiratory support in early stages of their disease [61,62].

## 6. Differential Diagnoses

Since myocarditis symptoms are not specific, other diseases can present in a similar clinical pattern [6,63]. In the newborn or infant, sepsis, hypoxia, severe dehydration, hypoglycaemia or anaemia must be ruled out. Other diagnoses should also be excluded, such as endocarditis and endocardial fibroelastosis. Genetic X-linked disorders, like Barth syndrome, as well as congenital structural heart lesions, such as critical coarctation of the aorta or anomalous origin of the left coronary artery from the pulmonary artery or even a cerebral arteriovenous malformation may also be present. In the older child, chronic tachyarrhythmia and pericarditis are two other differential diagnosed conditions to add to the previously listed.

As far as DCM is concerned, only one-third of the patients enrolled in a Paediatric Cardiomyopathy Registry study had a known cause of DCM at diagnosis [64]. On the subject of differential diagnoses, it is very important to consider familial DCM, since it comprises 14% of all paediatric DCM cases with a known cause and it carries a worse prognosis than other aetiologies [65,66,67]. Many mutations with autosomal dominant, recessive, X-linked, and mitochondrial inheritance patterns associated with the DCM phenotype have been identified [68]. Family cardiac screening may carry some advantages if done quickly, particularly the possibility of avoiding the need for the performance of other invasive and expensive diagnostic tools [65]. Other alternate diagnoses consistent with clinical presentation of symptomatic heart failure should be excluded, such as metabolic DCM (comprising 11% of paediatric DCM cases with a known cause), valvular and congenital heart disease (occurring in only 3% of paediatric DCM cases with a known aetiology), bronchiolitis, thyroid disease and chemotherapy-related cardiomyopathy [66,69,70,71]. Some genetic disorders, involving 26% of paediatric DCM cases with a known cause, like Emery–Dreifuss muscular dystrophy, Laing distal myopathy or Duchenne and Becker muscular dystrophy should be investigated in idiopathic DCM, after exclusion of all other identifiable causes [66,70].

## 7. Diagnosis and Treatment: A Triphasic Approach

Having in mind the triphasic framework of myocarditis, it is helpful to systematize its diagnostic and therapeutic strategies, which are considerably different for each stage of disease [38].

### 7.1. First phase: Viral Replication

#### 7.1.1. Diagnosis

Initially, the diagnosis of myocarditis is very dependent on clinical suspicion based on history and clinical picture of the disease, especially when cardiac involvement is apparent [6,7]. However, the wide range of clinical manifestations of paediatric myocarditis may easily transpire unnoticed by the clinician [59].

Definite diagnosis of viral myocarditis can only be proved by finding evidence of active viral infection, using histological or serological identification of virus [6,38]. EMB used to be required to define myocarditis according to the Dallas criteria [4]. Since it is not performed routinely in some centres, there are several non-invasive tests that can support the suspected diagnosis, something that is particularly important in paediatric patients [72].

The 12-lead electrocardiogram (ECG) is an easily available initial test [4]. Retrospective studies have found abnormal ECGs in 93% to 100% of paediatric patients with myocarditis [59,73]. However, a normal ECG does not exclude the possibility of the disease [57]. The most common findings in the acute phase of clinical myocarditis are sinus tachycardia with low-voltage QRS complexes and nonspecific T-wave changes, but these are not specific markers [4,6,21]. The ECG may also reveal ST-segment decrease or elevation, occasionally resembling a pattern seen in acute myocardial infarction [74,75]. Although commonly used as a screening tool, an ECG has the sensitivity of only 47% for the diagnosis of myocarditis in paediatric patients [76,77].

Echocardiography is still the most useful imaging technique to be performed in paediatric suspected cases to assess left ventricular structure, wall motion abnormalities, regional or global ventricular dysfunction and valvular insufficiency, since these abnormalities are often present in acute myocardial inflammation [7,10,78]. However, there are no specific echocardiographic features of myocarditis and they are usually insufficient to differentiate this disease from other forms of cardiomyopathy [6,51].

**Figure 2:**
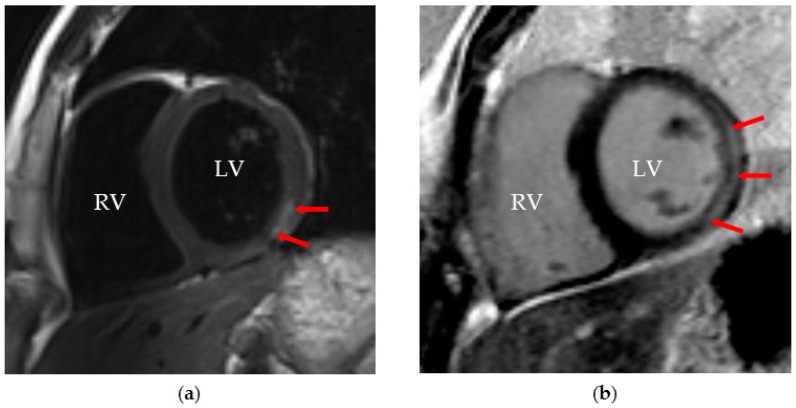
A 15-year-old boy with Epstein–Barr virus myocarditis: (**a**) a short-axis T2-weighted image demonstrating focal myocardial oedema (red arrows); and (**b**) a short-axis T1-weighted late gadolinium enhancement image (red arrows). RV, right ventricle; LV, left ventricle.

Cardiac MRI (cMRI) is proving to be the most attractive imaging tool for diagnosing myocardial inflammation and myocyte injury, especially if performed within 14 days of the beginning of symptoms, when the sensitivity for the diagnosis is higher (Figure 2) [6,79]. In addition to its unique potential for providing anatomic and morphologic information, cMRI has the ability to characterize tissue by measuring T1 and T2 relaxation times and spin densities, which are dependent on water content [80,81]. Detection of myocardial oedema, the initial change in myocardial tissue during the first phase of inflammation, is measured with T2-weighted imaging [51,80]. Gadolinium diethylenetriaminepentacetate (Gd-DTPA) is used as an extracellular contrast agent in contrast-enhanced cMRIs, and it distributes differently according to the type of cardiac tissue, inflamed or normal tissue [7]. Early enhancement in T1-weighted images obtained minutes after Gd-DTPA infusion may assess hyperaemia, but it is not the most specific finding for the diagnosis due to artefacts [82,83]. On the other hand, delayed Gd-DTPA enhancement suggests the presence of myocardial fibrosis and necrosis associated with myocarditis, lesions that are often histologically patchy in nature [7,84]. One cMRI approach to diagnose myocarditis is the Lake Louise criteria (LLC) [85]. The diagnosis is made if two out of three LLC criteria are met: (i) increased myocardial early Gd-DTPA enhancement ratio or absolute myocardial enhancement of ≥45% on T1-wheighted images; (ii) increased myocardial signal intensity on T2-weighted images (increased T2-ratio or regional increase in T2 signal intensity); and (iii) non-ischaemic lesions at late Gd-DTPA enhancement imaging [83]. Even though the LLC have shown some utility, particularly in patients with acute onset of symptoms, their qualitative nature and bad performance when applied in patients with heart failure and chronic symptoms has contributed to the search for novel quantitative T1 and T2 mapping techniques, including the quantification of extracellular volume [85]. A recent study performed in adult patients showed that myocardial T1 and T2 mapping and T1-derived extracellular volume fraction significantly improved the diagnostic accuracy of cMRI compared with conventional LLC [86]. By identifying areas of myocardial inflammation globally, cMRI offers an advantage in the diagnosis of this disease when compared to EMB and may even be used to direct it, consequently improving its diagnostic sensitivity [6,87]. However, cMRI with delayed Gd-DTPA enhancement alone has been reported to have a relatively low sensitivity in visualizing areas of myocarditis in adult patients, ranging from 27% to 95% [75]. Additionally, the use of cMRI in routine practice is limited by the lack of availability in most emergency settings and the need for general anaesthesia in young paediatric patients [88].

EMB is still the gold standard of diagnosing acute myocarditis even though in the past its routine clinical use for the diagnosis of myocarditis was not recommended [7,10]. The 2013 position statement of the European Society of Cardiology Working Group recommended that all patients with clinically suspected myocarditis should be considered for selective coronary angiography and EMB [89]. The role of EMB is undeniable, since immunohistochemistry and immunofluorescence techniques, viral DNA or RNA identification by PCR and real-time PCR amplification of viral genome, used in conjunction with standard EMB, provide an increase in diagnostic sensitivity [6,89]. However, EMB is not routinely performed to diagnose myocarditis [90]. The sensitivity of this diagnostic tool is limited due to sampling error and the rate of major complications associated with this procedure is variable, ranging from <1%, when performed in experienced centres, to 5% [91,92,93]. Some of the adverse events associated with EMB in paediatric patients may include atrial tachyarrhythmia, transient hypotension attributed to anaesthesia, transient ST-T wave changes, supraventricular tachycardia requiring electrical cardioversion, ventricular fibrillation and myocardial perforation [94].

#### 7.1.2. Treatment

Management of an early fulminant disease with cardiogenic shock is fundamentally supportive and is not aimed at the causative agent [7]. Paediatric patients with severe haemodynamic compromise related to myocarditis require rescue with aggressive support measures, including inotropic therapy and arrhythmia management, which may be life-saving [95]. Mechanical circulatory support devices such as ventricular assist devices (VADs) and extracorporeal membrane oxygenator (ECMO) should be considered early in patients who have severely compromised cardiac output despite optimal pharmacological treatment [4,96,97,98,99].

In initially stable patients or in the ones who survive the initial critical phase, therapy in the first stage of myocarditis may benefit from pharmacological eradication of the responsible viral agent in order to attenuate the cardiac injury [38]. However, direct antiviral therapies are only helpful in the small amount of cases in which the viral pathogen has established itself—like ganciclovir for CMV infection—or in the setting of a viral epidemic [6,100]. In this phase, it is extremely important to avoid both potentially damaging immunosuppression and non-specific antiviral procedures [6].

### 7.2. Second Phase: Autoimmune Activation

#### 7.2.1. Diagnosis

The autoimmune stage is diagnosed by EMB, which may show lymphocytic infiltration [6,38]. The original description of the histology of inflammation of the myocardium in patients with myocarditis was stated in the qualitative Dallas criteria as the presence of lymphocytic infiltrates in the myocardium associated with myocyte necrosis of non-ischaemic cause [4,101]. More recently, immunohistochemistry techniques have gained further acceptance in the detection of inflammation in EMBs, contributing to the increased number of EMB revealing myocarditis [5,10,102]. During EMB analysis, specific inflammatory cells can be distinguished by cluster differentiation (CD) [101]. Monoclonal antibodies to CD3 and CD68 or CD11 allow the detection and localization of T cells and activated macrophages, respectively [10]. CD20 stands for B-cells [101]. Human leukocyte antigen (HLA) can be used to detect HLA class II expression in professional antigen-presenting immune cells [10,37]. The addition of immunohistochemical criteria in the analysis of EMBs has made it possible to quantitatively define myocardial inflammation as focal and diffuse mononuclear infiltrates with >14 leucocytes/mm^2^ [5,101]. T cell subpopulations may include CD4 (helper), CD8 (suppressor) and CD45R0 (memory or activated T-cells) [101]. Having these subpopulations in mind, inflammation may be more specifically diagnosed by >7.0 CD3+ lymphocytes/mm^2^ and/or >35.0 CD11b+/Mac-1+ macrophages/mm^2^ [103].

Viral serologies may be abnormal as well [6]. However, IgG antibodies for cardiotropic viruses are often positive in the blood stream without associated cardiac involvement, a fact that limits its significance in the diagnostic process [6,104].

#### 7.2.2. Treatment

Since autoimmunity myocardial inflammatory processes may take place despite virus elimination, immunomodulatory therapies targeting the host immune system can be efficacious [4,7]. However, this topic remains controversial in the paediatric age group due to the reduced number of studies performed and the lack of respective controls [7]. Some administered immunomodulatory strategies include immunosuppression with corticosteroids, intravenous immunoglobulin (IVIG), azathioprine, and cyclosporine [105].

Corticosteroid treatment remains controversial when it comes to its benefit in children with myocarditis [106]. While some recent studies have shown improvement in left ventricular ejection fraction (LVEF) and symptoms among children treated with corticosteroids and/or other immunosuppressive agents, like cyclosporine, others found no differences in the final outcome [16,107,108,109]. The only aspect that seems to be commonly accepted is that immunosuppression with corticosteroids should not be used in the acute phase of myocarditis since it interferes with its natural eradication and, therefore, prolongs the disease [109].

IVIG has become part of routine immunomodulatory therapy for treating children with acute myocarditis at many centres, in a standard high-dose of 2 g/kg per 24 h [4,110]. There is a possibility that paediatric patients are more likely to respond to IVIG than adults due to a higher chance of a viral myocarditis [110]. Drucker et al. performed a retrospective study to assess the effect of a 2 mg/kg dose of IVIG therapy in 21 consecutive children with presumed acute myocarditis on survival and recovery of left ventricular function [111]. Their experience found that the use of high-dose IVIG was associated with improvements in recovery of left ventricular systolic function and a tendency toward better overall survival during the first year after presentation [10,111]. Nigro et al. reported a case of a seven-month-old girl with a severe myocarditis and persistent Parvovirus B19 DNA in the blood, who received IVIG therapy containing neutralizing antibodies specific to that virus [112]. The clinical improvement and viral clearing suggested the beneficial role of this therapeutic agent. A paediatric trial conducted by Bhatt et al. in India evaluated the efficacy of IVIG among 83 children (ages ranging from two months to 12 years) with suspected viral encephalitis and associated myocarditis [113]. They found a lower death rate among children receiving IVIG than in those who did not, and it is suggested that IVIG may be useful in the select group of children beyond the neonatal period who have the association of both viral diseases. Thus, a high risk of bias is present in this study, since it is not clear whether this benefit extends to children with myocarditis alone [110]. On the other hand, a retrospective analysis revealed that the treatment with IVIG provided no advantage in overall survival and time to recovery of normal left ventricular function in comparison to steroid therapy alone [114,115]. The question of IVIG role in paediatric myocarditis remains to be answered [114]. The insufficient evidence to support its routine use may require a prospective randomized study in order to truly assess the advantage of this treatment in this specific population [6,10,110].

Immunoadsorption is a therapeutic concept currently under discussion [6]. Its aim is to eliminate cardiotoxic autoantibodies together with cytokines, which have been associated with DCM [116,117]. Repetitive plasmapheresis sessions allow the removal of IgG primarily and little amount of IgA and IgM, and have been associated with improvement in cardiac function and reduction of oxidative stress [118,119,120]. In some studies, polyclonal IgG has been given at the end of the procedure, in order to provide an additional immunomodulatory effect [6]. The exact mechanism of action is still not known, but the benefit of this process may come from the removal of circulating autoantibodies, elimination of cytokines or unloading of the heart and the circulation by the temporary removal of proteins [119,121].

Jahns et al. have provided direct evidence of specific circulating β1-adrenoceptor antibodies [122,123]. This autoimmune attack, which also plays a causal role in DCM, can be eradicated with the elimination of the β-receptor antibody [6]. However, this pathophysiologically appealing concept lacks randomized clinical trials [6,119].

### 7.3. Third Phase: Dilated Cardiomyopathy

#### 7.3.1. Diagnosis

**Figure 3:**
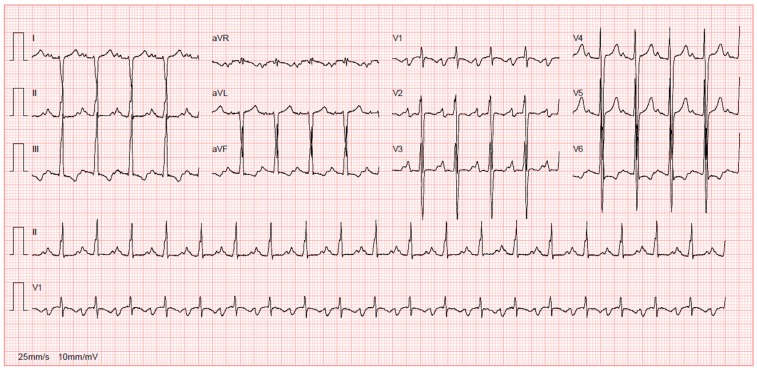
ECG of a 14-year-old girl with familial DCM showing sinus tachycardia, right axis deviation and nonspecific ST-wave changes.

**Figure 4:**
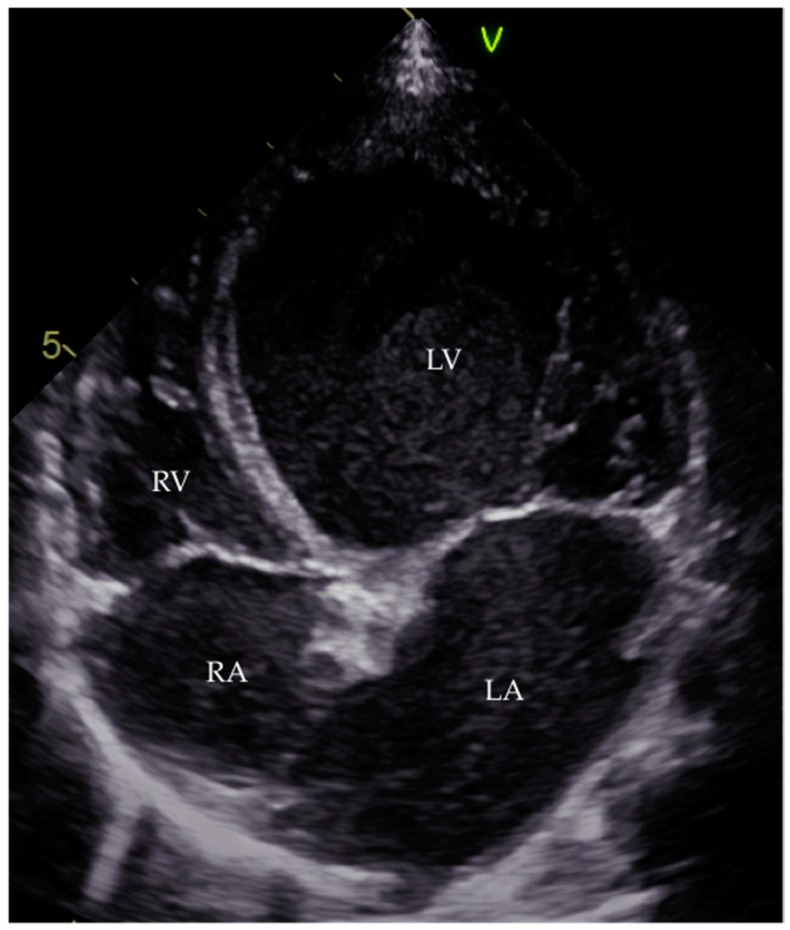
Four-chamber apical view echocardiogram of an 11-month-old boy with DCM. RA, right atrium; LA, left atrium; RV, right ventricle; LV, left ventricle.

This phase is identified by the usual imaging tools in order to exclude other causes of dilatation (Figure 3 and Figure 4) [6]. When it comes to echocardiography, it is important to acknowledge that even though the most frequent echocardiographic finding associated with myocarditis is a DCM phenotype of left ventricular dilatation and reduced ejection fraction, other parameters of ventricular remodelling may be present in histologically proven myocarditis, including regional hypertrophy and regional wall motion abnormalities [7,124]. The left ventricle of a patient with acute myocarditis may even lack significant dilatation [4].

#### 7.3.2. Treatment

Therapy after absence of both infectious and autoimmune stages is focused on prevention and reversal of the unfavourable remodelling process of the myocardium and reduction of haemodynamic stress by using angiotensin converting enzyme (ACE) inhibitors, β-blockers and spironolactone [6,37,38]. β-blockers like carvedilol can be cautiously introduced only in stable patients and the dose should be titrated slowly [125]. These agents must be stopped whenever inotropes are needed [121].

Anticoagulation with warfarin in DCM stage is difficult in young infants and many clinicians prefer to use aspirin in patients with severely compromised cardiac output or atrial dysrhythmias, in order to prevent the formation of mural thrombi [126,127]. However, aspirin and other non-steroidal anti-inflammatory agents have been associated with exacerbation of heart failure, probably due to the inhibition of prostaglandin synthesis, which ultimately may lead to a decrease in renal perfusion [121]. Their use is not recommended in adult patients with reduced left ventricular function according to the “2016 European Society of Cardiology Guidelines for the diagnosis and treatment of acute and chronic heart failure” [128]. Prophylactic aspirin should be reserved for paediatric patients with DCM who have not experienced any previous thromboembolic event or intracardiac thrombus, with LVEF between 25% and 30% or fractional shortening (FS) between 15% and 20%. Treatment should be stopped whenever systolic function improves [129].

Although paediatric myocarditis often improves within the first months of presentation, a minority of patients may progress to severe heart failure, which is unresponsive to optimal medical therapy, therefore requiring cardiac transplantation [7,10,21].

These patients need to be frequently monitored throughout their lives and a complete history, examination, and echocardiography should be undertaken [37].

## 8. Prognosis

Since a significant number of cases of myocarditis do not get diagnosed due to the unspecific symptoms, its true incidence and outcome remain difficult to characterize [7,61,97,130]. A lack of studies aiming to assess the prognosis of paediatric myocarditis adds to the difficulty and the extrapolation of outcomes from the adult to the paediatric population should not be assumed [97].

The main outcomes paediatric patients can experience include complete recovery, progression to DCM and death or transplantation [4,61,91]. According to Peta and co-workers’ 20-year study of 175 children with myocarditis, survival free from death or transplantation was 74% at one year, 65% at five years, 62% at 10 years, and 56% at 20 years. At 15 years after diagnosis, 60% of the participants remained free from transplantation, of whom 69% achieved echocardiographic normalization of left ventricular function, 96% were free of cardiac symptoms, 80% were not receiving long-term medical therapy, and 4% had an implantable cardioverter-defibrillator [131]. Another study including 222 paediatric patients with DCM due to myocarditis revealed a five-year rate of freedom from transplantation of 81% and an estimated survival of 92% at one year and 90% at two and five years [119]. In an apparently paradoxical way, patients with fulminant myocarditis have a more favourable long-term survival than patients with acute myocarditis [61]. The initial clinical presentation of abrupt-onset haemodynamic instability experienced by these patients requires an aggressive intensive care management, which probably accounts for the better prognosis [4,61]. According to a Paediatric Cardiomyopathy Registry study, most patients experience normalization of ventricular size and systolic function within several months and it is more likely to occur in patients with a normal left ventricular diastolic diameter or with a greater left ventricular posterior wall thickness [132].

## 9. New Perspectives

Throughout the past few decades, our understanding of the molecular basis of myocarditis has grown, which has allowed the development of many diagnostic and therapeutic innovations [90,118,132].

New molecular technologies have recently been used to characterize upregulations in micro-RNA profiles during the pathological progression of myocarditis [76]. Additionally, prevention of remodelling of the myocardium by matrix metalloproteinases and identification of potent novel antivirals and biological medications seem to be appealing treatment concepts and are currently on the radar of some recent studies [118]. These promising avenues may lead to future development of interesting and accurate targets for the diagnosis and treatment of myocarditis in paediatric patients.

## 10. Conclusions

Myocarditis is a potentially life-threatening disease often clinically disguised as a benign one. Its tendency to occur in young patients makes it one of the most frequent causes of DCM in this age group. The amount of research made in experimental animal models in the last few decades has allowed a better understanding of this disease, particularly its triphasic nature. However, assessing an unambiguous prompt diagnosis of myocarditis and correctly staging it probably remains the hardest challenge for clinicians, as this information is useful to initiate a specific treatment and improve the outcome. Therefore, further clinical trials are needed in a quest for novel efficient diagnosis and treatment strategies, which could eventually lead to a reduction in paediatric morbidity and mortality.
